# Absent and abundant MET immunoreactivity is associated with poor prognosis of patients with oral and oropharyngeal squamous cell carcinoma

**DOI:** 10.18632/oncotarget.7534

**Published:** 2016-02-20

**Authors:** Maria J. De Herdt, Stefan M. Willems, Berdine van der Steen, Rob Noorlag, Esther I. Verhoef, Geert J.L.H. van Leenders, Robert J.J. van Es, Senada Koljenović, Robert J. Baatenburg de Jong, Leendert H.J. Looijenga

**Affiliations:** ^1^ Department of Othorhinolaryngology and Head and Neck Surgery, Erasmus MC, University Medical Center Rotterdam, Rotterdam, The Netherlands; ^2^ Department of Pathology, University Medical Center Utrecht, Utrecht, The Netherlands; ^3^ Department of Oral and Maxillofacial Surgery, University Medical Center Utrecht, Utrecht, The Netherlands; ^4^ Department of Pathology, Erasmus MC, University Medical Center Rotterdam, Rotterdam, The Netherlands

**Keywords:** oral and oropharyngeal squamous cell carcinoma, C-terminal MET, immunohistochemistry, antibody validation, prognosis

## Abstract

Although the receptor tyrosine kinase (RTK) MET is widely expressed in head and neck squamous cell carcinoma (HNSCC), its prognostic value remains unclear. This might be due to the use of a variety of antibodies and scoring systems. Here, the reliability of five commercial C-terminal MET antibodies (D1C2, CVD13, SP44, C-12 and C-28) was evaluated before examining the prognostic value of MET immunoreactivity in HNSCC. Using cancer cell lines, it was shown that D1C2 and CVD13 specifically detect MET under reducing, native and formalin-fixed paraffin-embedded (FFPE) conditions. Immunohistochemical staining of routinely FFPE oral SCC with D1C2 and CVD13 demonstrated that D1C2 is most sensitive in the detection of membranous MET. Examination of membranous D1C2 immunoreactivity with 179 FFPE oral and oropharyngeal SCC – represented in a tissue microarray – illustrated that staining is either uniform (negative or positive) across tumors or differs between a tumor's center and periphery. Ultimately, statistical analysis revealed that D1C2 uniform staining is significantly associated with poor 5-year overall and disease free survival of patients lacking vasoinvasive growth (HR = 3.019, *p* < 0.001; HR = 2.559, *p* < 0.001). These findings might contribute to reliable stratification of patients eligible for treatment with biologicals directed against MET.

## INTRODUCTION

Head and neck cancers (HNCs) are a diverse group of malignant tumors that arise in various anatomical localizations of the upper aerodigestive tract [1]. With an incidence of more than 680,000 cases worldwide (42,913 in Western Europe), it is the seventh most common cancer [2]. Moreover, 90 to 95% percent of HNCs are squamous cell carcinoma (HNSCC).

Despite the fact that numerous treatment platforms are available [3–5], relative 5-year survival rates remain poor for patients presenting with locoregionally advanced and recurrent and/or metastatic HNSCC [6–8]. The current standard of care for patients diagnosed with advanced, unresectable HNSCC is concurrent chemoradiation (CRT) [9]. Nevertheless successful, CRT is associated with substantial toxicity impeding its advances [10]. Consequently, translational research has focused on the application of biologicals in the treatment of advanced HNSCC [10]. This effort ultimately led to the approval of cetuximab, a monoclonal antibody directed against the epidermal growth factor receptor [1, 10]. Although adding cetuximab to radiotherapy (RT) improves locoregional control and reduces mortality compared to RT alone [11], there is no – prospective – evidence that the use of cetuximab plus RT in advanced HNSCC outperforms CRT [12, 13]. However, adding cetuximab to chemotherapy in patients with recurrent and/or metastatic HNSCC does provide a small though significant survival benefit compared to chemotherapy alone [14, 15]. To further improve treatment options for patients diagnosed with advanced and recurrent and/or metastatic HNSCC, the search for additional relevant molecular targets continues [16]. One molecular target of interest is the receptor tyrosine kinase MET [1, 10, 16].

MET is synthesized as a partially glycosylated 170 kDa single-chain intracellular precursor, which undergoes cleavage and further glycosylation to yield a mature, cell surface-associated 190 kDa disulphide linked heterodimer consisting of an extracellular 50 kDa a-chain and a transmembrane 145 kDa b-chain [17]. MET is predominantly expressed on the surface of epithelial cells and is activated by its stromal ligand, the hepatocyte growth factor/scatter factor (HGF/SF) [18]. Signaling via this receptor-ligand pair initiates the program of invasive growth, which is essential for physiological processes such as embryogenesis, tissue regeneration and wound healing as well as the pathological process of cancer cell invasion.

Deregulated HGF/SF-MET signaling has been implicated in many human solid cancers [19], which has led to the development of biologicals that target MET [19]. Today's challenge lies in the reliable stratification of patients eligible for treatment with MET inhibitors [19]. Although MET is abundantly expressed and acts as an orchestrator of invasive growth in HNSCC [20], its role as a prognostic factor remains unclear [21–30]. This might be due to the use of a variety of antibodies resulting in varying staining patterns and scoring systems. Moreover, several antibodies showed significant lot-to-lot variability in terms of specificity, sensitivity and staining patterns [31, 32].

Using the Rimm Lab Algorithm for antibody validation [33] as guidance, this study investigates the specificity and sensitivity – for single lots – of five commercial antibodies directed against the C-terminus of MET (i.e., D1C2, CVD13, SP44, C-12 and C-28) under reducing, native and formalin-fixed paraffin-embedded (FFPE) conditions using a panel of well characterized cell lines – of which one was silenced for *MET* using a siRNA. Next, the antibodies that behaved reliably across all examined conditions (i.e., D1C2 and CVD13) were used to explore MET immunoreactivity across whole tissue sections of a selection of oral SCC.

Finally, using the antibody that is most sensitive in the detection of membranous MET (i.e., D1C2), it was examined whether MET immunoreactivity is associated with the survival of 179 patients diagnosed with oral and oropharyngeal SCC of whom long-term clinico-pathological follow-up was available.

## RESULTS

### Comparison of commercial antibodies directed against the C-terminus of MET

As a guide, the Rimm Lab Algorithm for antibody validation [33] was used to check the specificity and sensitivity of the five purchased C-terminal MET antibodies (i.e., D1C2, CVD13, SP44, C-12 and C-28). In short, the algorithm states that the performance of antibodies should be as expected under all examined – reducing, native and FFPE – conditions in order to be found reliable. To properly asses the validity of the examined antibodies, their specificity and sensitivity was evaluated per examined condition based on the results described below. The details and properties of the used antibodies are described in the Materials and Methods section, paragraph antibodies (Table 1).

**Table 1:** Properties of the purchased MET antibodies

Clone	Catalog#	Company	Species	Clonality	Isotype	Reactivity	Antigen region	Immunogen	Peptide length and amino acid mapping region
D1C2	8198	Cell Signaling Technology^®^	Rabbit	Mono	IgG	Human	C-terminus of Human MET	Peptide	Mapping to residues near the C-terminus of Human MET. Peptide length, specific amino acid region & MET isoform are proprietary information.
SP44*	M3440	Spring™ Bioscience	Rabbit	Mono	IgG	Human	C-terminus of Human MET	Peptide	Mapping to residues near the C-terminus of Human MET. Peptide length, specific amino acid region & MET isoform are proprietary information.
CVD13*	71-8000	Invitrogen™	Rabbit	Poly	IgG	Human	C-terminus of Human MET	Peptide	Mapping within the last 30 C-terminal amino acids of Human MET (Accession: NP_001120972.1). Peptide length is proprietary information.
C-12	Sc-10	Santa Cruz Biotechnology, Inc.	Rabbit	Poly	IgG	Human	C-terminus of Human MET	Peptide	15-25 amino acid long peptide mapping within the last 50 C-terminal amino acids of Human MET (Accession: NP_000236.2).
C-28	Sc-161	Santa Cruz Biotechnology, Inc.	Rabbit	Poly	IgG	Human, Mouse & Rat	C-terminus of Human MET	Peptide	15-25 amino acid long peptide mapping within the last 50 C-terminal amino acids of Human MET (Accession: NP_000236.2).

* Information concerning this clone might be discrepant with information published on the internet, since the corresponding datasheet has been revised over time.

With the exception of the peptide length and amino acid mapping regions for CVD13, C-12 and C-28, the information summarized in the table above is extracted from the datasheets provided with the antibodies. The detailed information concerning the peptide length and/or amino acid mapping regions for CVD13, C-12 and C-28, is obtained through direct communication (telephone and e-mail) with the technical support services of Invitrogen™ (CVD13) and Santa Cruz Biotechnology, Inc. (C-12 and C-28).

**Figure 1:**
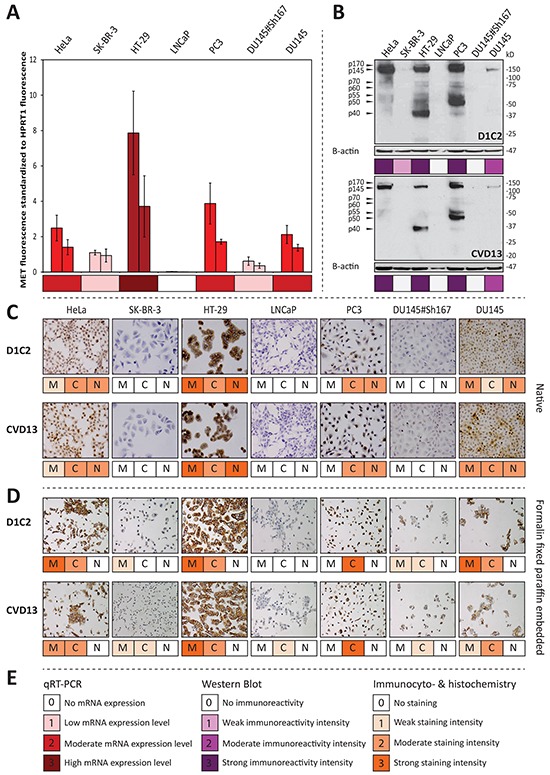
D1C2 and CVD13 immunoreactivity in respect to MET *mRNA* expression levels across the antibody validation cell line panel **A.** qRT-PCR results showing average *MET* fluorescence standardized to average *HPRT1* fluorescence and accompanying standard deviations (*n* = 3), which are derived from biological duplicates of all cell lines included in the antibody validation panel. **B.** immunoreactivities observed with western blotting. For further information concerning the MET specific protein bands, the reader is referred to Supplementary Table S2. **C.** membranous (M), cytoplasmic (C) and nuclear (N) immunocytochemical reactivity **D.** membranous (M), cytoplasmic (C) and nuclear (N) immunohistochemical reactivity. **E.** legend for observed mRNA expression levels, western blot immunoreactivities and immunocyto- & immunohistochemical reactivities.

To establish a baseline for MET expression, *MET* mRNA expression levels were determined in the MET antibody validation cell line panel (Supplementary Table S1; Materials and Methods section, paragraph MET antibody validation cell line panel and culture conditions) by means of qRT-PCR. Although *MET* mRNA expression levels vary markedly between the cell lines (Figure 1A), ranging from very low (LNCaP) to high (HT-29), none of the cell lines are completely devoid of *MET* mRNA (i.e., truly negative). It should be mentioned here that we depicted LNCaP as negative for *MET* mRNA expression in Figure 1A because standardized *MET* fluorescence levels in this cell line are so low that they cannot be observed in the presented bar chart.

Before assessing the specificity of the antibodies under reducing conditions, it was assumed that cell lines with low *MET* mRNA expression levels will show no or weak immunoreactivity with bands migrating as MET protein products and C-terminal fragments (Supplementary Table S2). The immunoblots generated with D1C2 and CVD13 (Figure 1B) show band patterns that are specific for MET protein products and C-terminal fragments. Furthermore, the observed intensities are in line with the established *MET* mRNA expression levels. Moreover, in contrast to its parental cell line (DU145), no immunoreactivity was detected in the *MET* silenced cell line (DU145#Sh167). When comparing the intensities of the blots generated with D1C2 and CVD13 (Figure 1B), D1C2 shows a stronger immunoreactivity compared to CVD13. This is especially true for the p70^MET^ and p60^MET^ C-terminal fragments observed in HeLa, HT-29 and PC3. In contrast, the immunoblots generated with SP44 and C-12 illustrate that these antibodies are not reliable in detecting of MET protein products and C-terminal fragments (Supplementary Figures S1A & S1B). Although the immunoblot generated with SP44 (Supplementary Figure S1A) only shows immunoreactivity with bands migrating as the expected protein products (Supplementary Table S2), the antibody's performance under reducing conditions was evaluated as nonspecific because of the strong immunoreactivity with a 90 kDa protein product in SK-BR-3 and LNCaP - both cell lines showing low *MET* mRNA expression levels. C-12's performance under reducing conditions (Supplementary Figure S1B) was also evaluated as nonspecific, since it shows immunoreactivity with an unexpected 15 kDa protein product in LNCaP, PC3, DU145 and DU145#Sh167. C-12's nonspecific behavior was further corroborated by moderate immunoreactivity with a 55 kDa protein product in the *MET* silenced cell line (DU145#Sh167). The immunoblot generated with C-28 was found too poor to evaluate (Supplementary Figure S1C). Taking everything into consideration, it is concluded that only D1C2 and CVD13 specifically detect – at least partly – the expected MET protein products and C-terminal fragments (Supplementary Table S2) under reducing conditions. Yet, CVD13 is less sensitive compared to D1C2 (Figure 1B).

Under native conditions, immunoreactivities were observed in the nucleus, cytoplasm and at the membrane. Separate scores were given for each cellular compartment. Analogous to the assumptions made under reducing conditions, it was assumed that cell lines with low *MET* mRNA expression levels will show no or weak immunoreactivity irrespective of the cellular location. This is not the case for C-12 and C-28, which are therefore considered to be nonspecific in the detection of MET (Supplementary Figure S2). However, this is the case for D1C2, CVD13 and SP44 which are therefore considered to be specific in the detection of MET under native conditions. The specific behavior of these three antibodies is further supported by the absence of immunoreactivity in the *MET* silenced cell line (DU145#Sh167). In contrast to D1C2 and CVD13, SP44 shows weak immunoreactivity with SK-BR-3 and LNCAP making it the most sensitive antibody under native conditions (Figure 1C, Supplementary Figure S2).

Also under FFPE conditions, immunoreactivities were observed in the nucleus, cytoplasm and at the membrane. Again, separate scores were given for each cellular compartment and – similar to the assumptions made under native conditions – it was assumed that cell lines with low *MET* mRNA expression levels will show no or weak MET immunoreactivity irrespective of the cellular location. This is not the case for SP44, C-12 and C-28, which are therefore considered to be nonspecific in the detection of MET under FFPE conditions (Supplementary Figure S3). However, this is the case for D1C2 and CVD13, which are therefore considered to be specific in the detection of MET in FFPE cells. This is further supported by the weak immunoreactivity observed in the *MET* silenced cell line (DU145#Sh167). Comparing the staining intensities obtained with D1C2 and CVD13 per subcellular localization, reveals that D1C2 has a higher sensitivity for membranous MET and that CVD13 has a higher sensitivity for cytoplasmic MET in FFPE cells (Figure 1D).

To verify whether the latter observation holds true in a diagnostic setting, four routinely processed FFPE oral SCC were stained with D1C2 and CVD13. Again, D1C2 shows a higher sensitivity for membranous MET and CVD13 shows a higher sensitivity for cytoplasmic MET. This is especially true for immunoreactivities observed with salivary gland ducts and cancer cells (Supplementary Figures S4A & S4C).

**Figure 2:**
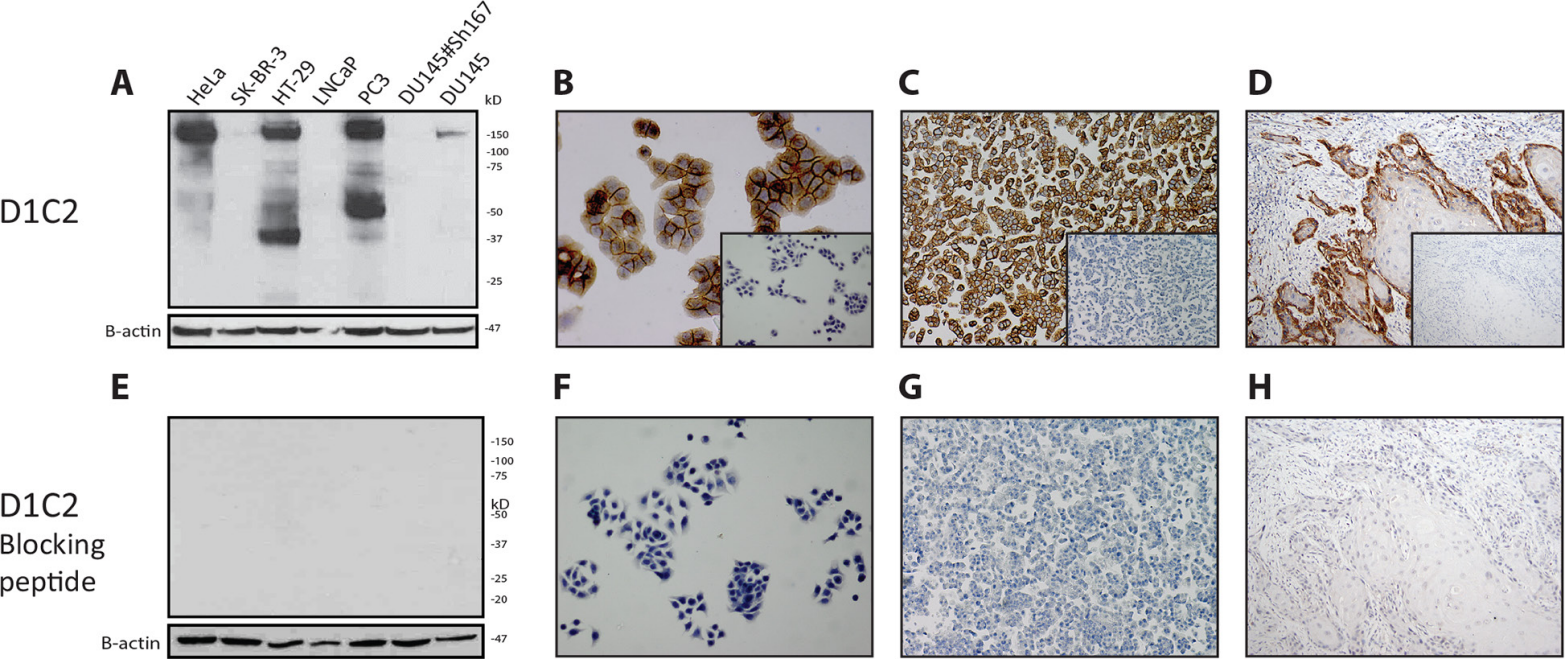
Demonstration of the complementarity between D1C2 and its blocking peptide After incubation of D1C2 with its blocking peptide, the complementarity of these reagents was checked under reducing, native and FFPE conditions by means of **E.** western blotting (antibody validation cell line panel). **F.** immunocytochemistry (HT-29). **G & H.** immunohistochemistry (HT-29 & representative oral SCC). **A through D.** Corresponding D1C2 immunoreactivities. Negative controls – of the latter immunocyto- & immunohistochemistry experiments – are depicted in the inlays.

The specificity of D1C2 was corroborated for all examined conditions by performing western blot analysis, immunocytochemistry and immunohistochemistry on all examined cell lines and cancers after pre-incubating the antibody with the peptide that was used for its generation (Figure 2).

Considering the made assumptions, the results indicate that SP44, C-12 and C-28 are not specific in the detection of MET protein product and fragments across all examined conditions and are therefore considered to behave nonspecifically. In contrast, D1C2 and CVD13 are specific across all examined conditions. In comparison to one another, D1C2 is slightly more sensitive in the detection of p170^MET^ and p145^MET^ under reducing conditions. Under native and FFPE conditions, D1C2 is more sensitive in the detection of membranous MET and CVD13 is more sensitive in the detection of cytoplasmic MET. Furthermore, the complementarity between D1C2 and the peptide that was used for its generation was confirmed under all investigated conditions.

### Evaluation of MET immunoreactivity in a cohort of oral and oropharyngeal SCC, a scatter plot based analysis

To study MET immunoreactivity in a series of oral and oropharyngeal SSC, a TMA was stained with the antibody that is most sensitive in the detection of membranous MET (i.e., D1C2). With cores taken from the center and periphery, the design of the TMA allows to explore the behavior of MET immunoreactivity across cancer surfaces. Further details concerning the design of the TMA are described in the Materials and Methods section, paragraph patient material.

Before assessing the behavior of MET immunoreactivity across cancer surfaces, the heterogeneity of MET immunoreactivity across cores sampled in the center and periphery was evaluated using the ICC. The ICC for the center cores was 0.930 (0.905, 0.949) and the ICC for the periphery cores was 0.894 (0.851, 0.926) indicating almost perfect agreement between cores from the same cancer region.

Evaluation of membranous MET immunoreactivity in both cancer regions (center and periphery) was possible in 183 (76.3%) cases, of which 4 (2.2%) oropharyngeal SCC were HPV-16 positive. Seen this low number, the HPV-16 positive oropharyngeal SCC were excluded from further analysis. The baseline characteristics of the remaining cancers are indicated in Table 2. The behavior of MET immunoreactivity across the remaining 179 cancer surfaces, was visualized by displaying corresponding percentages of MET positive – moderate (2) to strong (3) immunoreactivity (Materials and Methods) – cancer cells in the center and periphery as data points in a scatter plot (Supplementary Figure S5A). The result illustrates that data points are scattered across the entire chart area, indicating that the amount of MET immunoreactivity is either constant across the cancer (uniform negative or positive staining) or differs between the tumor center and periphery (variable staining).

**Table 2:** Summary of baseline characteristics

Characteristic	No. of patients	
	#	%
Sex		
Male	114	63.69
Female	65	36.31
Age at diagnosis (years)		
Mean (range)	62.97 (34 – 87)	
Smoking		
No	60	33.52
Yes	117	65.36
*Missing*	2	1.12
Alcohol		
No	81	45.25
Yes	96	53.63
*Missing*	2	1.12
Site		
Oral cavity	157	87.71
Oropharynx	22	12.29
Cancer stage*		
I	17	9.50
II	31	17.32
III	42	23.46
IV	89	49.72
Infiltration depth		
<4.0 mm	9	5.03
≥4.0 mm	170	94.97
Differentiation grade		
Good-moderate	141	78.77
Poor	38	21.23
Bone invasion		
Absent OR no bone present	140	78.21
Present	39	21.79
Growth pattern		
Cohesive	36	20.11
Non-cohesive	142	79.33
*Missing*	1	0.56
Perineural invasion		
Absent	98	54.75
Present	73	40.78
*Missing*	8	4.47
Vasoinvasive growth		
Absent	136	75.98
Present	39	21.79
*Missing*	4	2.23
Extranodal growth		
Absent OR pN0	124	69.27
Present	54	30.17
*Missing*	1	0.56
Treatment		
Surgery	50	27.93
Surgery and (chemo)radiotherapy	128	71.51
*Missing*	1	0.56

* Based on pTNM.

### Association between MET staining patterns and prognosis

To establish whether there is a relation between the pattern of MET immunoreactivity (uniform negative, uniform positive or variable staining) and survival, each data point in the scatter plot was labelled with the 5-year OS or DFS status of the corresponding patient (Supplementary Figures S5B & S5C). The result reveals that events (red dots) cluster in the lower left and upper right corners of both scatter plots. This nonrandom distribution of events provided the basis to assign one of the three defined staining patterns to each point in the scatter plot. Therefore, it was assumed that the cluster of points in the lower left corner of the scatter plot represents cancers with uniform negative MET staining patterns. Similarly, it was assumed that the cluster of points in the upper right corner of the scatter plot represent cancers with uniform positive MET staining patterns. Furthermore, it was assumed that the points residing outside the observed clusters represent cancers with variable MET staining patterns. Exact boundaries were set for the two observed clusters – representing uniform staining – in such a way that the relative number of events (i.e. OS or DFS) within them is higher compared to the relative number of events outside them. The boundary for the uniform negative staining cluster is < 10% MET immunoreactivity in the center and periphery. The boundary for the uniform positive staining cluster is > 75% MET immunoreactivity in the center and periphery (Supplementary Figures S5B & S5C).

**Figure 3:**
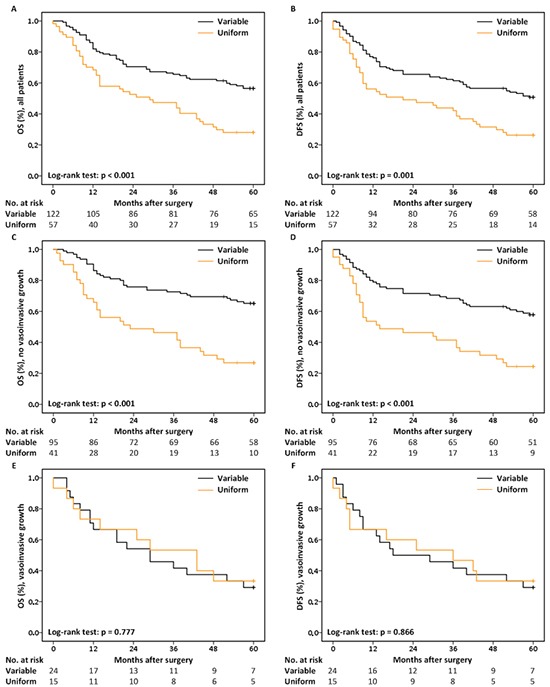
Kaplan-Meier curves and the No. of patients at risk **A.** 5-year OS & **B.** 5-year DFS for all patients, stratified by MET staining pattern. **C.** 5-year OS & **D.** 5-year DFS for patients lacking histological signs of vasoinvasive growth, stratified by MET staining pattern. **E.** 5-year OS & **F.** 5-year DFS for patients with histological signs of vasoinvasive growth, stratified by MET staining pattern.

Univariable analysis revealed that patients showing the variable staining pattern perform significantly better than patients showing either uniform staining patterns (negative or positive) in terms of 5-year OS (HR = 2.188, *p* < 0.001) and DFS (HR = 1.974, *p* = 0.001) (Figure 3A & 3B; Supplementary Figure S5D & S5E). Besides MET staining pattern, clinical and histological T and N-stage, differentiation grade, vasoinvasive growth and extranodal growth are significantly associated with poor 5-year OS (Supplementary Table S3). The same applies for: age at diagnosis, clinical and histological T and N-stage and extranodal growth with respect to 5-year DFS (Supplementary Table S3).

Multivariable Cox regression analyses revealed an effect of MET staining pattern on survival that is dependent on the status of vasoinvasive growth (Supplementary Tables S4 & S5). Where MET staining pattern significantly influences 5-year OS (HR = 3.019, *p* < 0.001; Figure 3C) and DFS (HR = 2.559, *p* < 0.001; Figure 3D) in patients lacking signs of vasoinvasive growth (*n* = 136), MET staining pattern has no effect on survival in patients with vasoinvasive growth (*n* = 39; Figure 3E & 3F).

Based on these data, further multivariable Cox regression analyses were restricted to the subgroup of patients lacking histological signs of vasoinvasive growth. The best multivariable Cox regression model for 5-year OS includes: MET staining pattern, clinical N-stage and extranodal growth, while the final model for 5-year DFS includes: MET staining pattern and extranodal growth (Table 3). In short, these results demonstrate that MET uniform staining patterns, either negative or positive, remain independently associated with the poor prognosis of patients lacking histological signs of vasoinvasive growth after adjusting for other factors known to be associated with a poor outcome of HNSCC.

**Table 3:** Explanatory variables significantly associated with 5-year OS and DFS

Variable		HR	95% CI	*p*-value
5-year OS	MET uniform staining pattern	3.475	2.081 - 5.801	< 0.001
	cN1-3	2.023	1.120 - 3.653	0.019
	Extranodal growth	4.207	2.229 - 7.942	< 0.001
5-year DFS	MET uniform staining pattern	2.923	1.797 - 4.756	< 0.001
	Extranodal growth	5.624	3.306 - 9.566	< 0.001

Results of the optimal multivariate Cox proportional hazards regression models including only patients lacking histological signs of vasoinvasive growth. Abbreviations: OS, Overall Survival; DFS, Disease Free Survival; HR, Hazard Ratio; CI, Confidence Interval. Bold values highlight statistical significance.

## DISCUSSION

The receptor tyrosine kinase MET is expressed in the majority of HNSSC [20], making it an interesting target for therapy [19]. Although several biologicals against MET have been developed, there are no guidelines concerning the stratification of patients eligible for treatment with MET inhibitors [19].

Despite MET's established role as a facilitator of invasive growth in HNSCC [20], its status as a prognostic factor remains unclear [21–30]. A possible explanation might be the use of a variety and potentially unreliable antibodies. Therefore, we investigated the specificity and sensitivity of single batches of five commercial antibodies directed against the C-terminus of MET under reducing, native and FFPE conditions before establishing the receptor's influence on the survival of patients with oral and oropharyngeal SCC.

Two out of five antibodies, D1C2 and CVD13, specifically detected MET across all examined conditions, confirming the need for biomarker validation and testing as discussed by Hayes *et al.* [34]. At this point, it should be stressed that the observed specificity of D1C2 and CVD13 should not be taken for granted, since the reliability of antibodies is known to vary between manufacturing lots. Consequently, one must always verify the specificity of D1C2 and CVD13 with changing lot numbers before using them in a research and/or clinical environment [33].

Besides the precursor (p170^MET^) and the b-chain (p145^MET^), D1C2 and CVD13 detected five additional MET C-terminal protein fragments (p40^MET^, p55^MET^, p50^MET^, p60^MET^ and p70^MET^) under reducing conditions. This finding is not unexpected as MET is subject to proteolysis [35]. Under conditions of stress, MET is cleaved by caspases leading to the generation of a proapoptotic intracellular 40 kDa C-terminal fragment, referred to as p40^MET^[36, 37]. Also, independently of ligand stimulation, MET can be cleaved within its extracellular juxtamembrane domain by membrane metalloproteases. This leads to the generation of a soluble MET N-terminal fragment which is shed into the extracellular space [35] – a process referred to as ectodomain shedding [38] – and a membrane-anchored C-terminal fragment (MET-CTF) of 55 kDa, referred to as p55^MET^ [39]. These specific MET-CTFs (55 kDa) can be processed through direct lysosomal degradation [39] or are cleaved at the membrane by the g-secretase complex, a process known as presenilin-regulated intramembrane proteolysis. The generated 50 kDa intracellular domain of MET (MET-ICD), referred to as p50^MET^, is subsequently released into the cytosol and degraded by the proteasome [40]. Finally, the observation that the rate of MET ectodomain shedding – in terms of the number of N-terminal fragments – increases with increasing malignant behavior of breast cancer cells [38], provides an explanation for the p70^MET^ and p60^MET^ C-terminal fragments observed in HeLa, HT-29 and PC3 using D1C2 under reducing conditions. More specifically – among the observed shed N-terminal fragments – two fragments were observed of 75 and 85 kDa. These specific N-terminal fragments – theoretically – give rise to MET-CTFs of 70 and 60 kDa.

Under native conditions, MET immunoreactivity – obtained with D1C2 and CVD13 – was observed in the nucleus, in the cytoplasm and at the membrane. This finding is not unexpected since MET and/or MET C-terminal fragments have been observed at each of these cellular locations [41–44]. In contrast, MET immunoreactivity was only observed in the cytoplasm and at the membrane of FFPE cells indicating that the nuclear epitope cannot be detected by these two antibodies under FFPE conditions. When comparing the membranous and cytoplasmic immunoreactivities obtained under FFPE conditions, the observed intensities indicate that CVD13 was most sensitive in the detection of cytoplasmic MET and D1C2 was most sensitive in the detection of membranous MET in FFPE cancer cells and tissues.

Because antibodies as well as a selection of tyrosine kinase inhibitors can target the receptor when it is located at the membrane [19], membranous MET immunoreactivity was further investigated in a series of oral and oropharyngeal SCC using D1C2. It was observed that membranous MET immunoreactivity is either constant across the cancer (uniform negative or positive staining) or differs between the tumor center and periphery (variable staining).

Before discussing the results obtained with survival analysis, we would like to stress that we realize that using boundaries for survival analysis based on biology is not indisputable. Therefore, efforts are needed – in the future – to validate the relation between MET staining patterns and survival. Hopefully, such efforts will result in a standardized scoring system for MET immunoreactivity that is applicable in a routine diagnostic setting.

Univariable survival analysis revealed that patients with cancers showing the variable staining pattern perform significantly better than patients showing either uniform staining patterns (negative or positive) in terms of 5-year OS and DFS. The significant association between uniform positive MET staining and poor survival is not unexpected, because MET is a known orchestrator of invasive growth [18, 20, 45, 46]. Although counterintuitive, the significant association between uniform negative MET staining and poor survival is not illogical as MET is expressed on the surface of epithelial cells under physiological conditions [18], which is corroborated by our results showing strong MET immunoreactivity with salivary glands ducts. Therefore, it is hypothesized that a tight balance exists between the amount of MET protein and tissue homeostasis. The observation that both low and high expression levels of ERBB2 – also a RTK involved in many epithelial cancers [47] – are associated with the poor prognosis of primary breast cancers [48], illustrates that our finding does not stand completely on its own.

In addition to MET staining pattern, the prognostic value of clinico-pathological characteristics known to be associated with the survival of patients with HNSCC [49] was examined. The results revealed that clinical and histological T and N-stage, differentiation grade, vasoinvasive growth and extranodal growth are significantly associated with poor 5-year OS. With the exception of differentiation grade and vasoinvasive growth, the same parameters were also significantly associated with poor 5-year DFS. Subsequent multivariable analysis revealed an effect of MET staining pattern on survival that is dependent on the status of vasoinvasive growth. Where MET staining pattern significantly influences 5-year OS and DFS in patients lacking signs of vasoinvasive growth, it has no effect on survival in patients with vasoinvasive growth. Since other studies describe that the presence of vasoinvasive growth is significantly associated with cancer recurrence and poor prognosis of patients diagnosed with HNSSC [50, 51], we hypothesize that the effect of MET staining pattern on survival is subservient to that of vasoinvasive growth. Therefore, further multivariable survival analysis was restricted to patients lacking signs of vasoinvasive growth. Since MET staining pattern significantly contributes to the final multivariable models for 5-year OS and DFS, we suggest that MET staining pattern might be of added value in treatment decision-making for patients lacking signs of vasoinvasive growth.

In conclusion, this study shows that using a specific and sensitive antibody directed against the C-terminus of MET, both scarce and abundant membranous MET immunoreactivity are significantly associated with poor survival rates of patients with oral and oropharyngeal SCC lacking signs of vasoinvasive growth. These findings might contribute to reliable stratification of patients eligible for treatment with biologicals directed against MET. Yet, whether patients suffering from cancers showing abundant membranous MET immunoreactivity could benefit from treatment with MET inhibitors needs further investigation.

## MATERIALS AND METHODS

### Antibodies

Western blot analysis, immunocyto- and immunohistochemistry were performed using five commercial antibodies directed against the C-terminus of MET, specifically D1C2 (Cell Signaling Technology^®^; Leiden, The Netherlands), CVD13 (Life Technologies™/Invitrogen™; Bleiswijk, The Netherlands), SP44 (Spring™ Bioscience; Pleasanton, CA 94566, USA), C-12 and C-28 (Santa Cruz Biotechnology, Inc.; Heidelberg, Germany). Additional property information on the antibodies is indicated in Table 1.

### Ethics statement

Human tissues and patient data were used according to “The Code for Proper Secondary Use of Human Tissue” and “The Code of Conduct for the Use of Data in Health Research” as stated by the Federation of Dutch Medical Scientific Societies (Federa FMVV, updated 2011).

### Patient material

Patients with histologically confirmed oral or oropharyngeal SCC – whose primary treatment was surgery – were included in this study. Patients diagnosed with synchronous primary cancers or previous malignancies in the head and neck region were excluded. Human papillomavirus type 16 (HPV-16) status was determined for all oropharyngeal SCC by means of the algorithm described by Smeets *et al.* [52] using the methods described by van Kempen *et al.* [53].

To examine MET immunohistochemical reactivity in routinely processed FFPE primary oral SCC, representative tissue blocks of four cancers were randomly collected from the archives of the department of pathology of the Leiden University Medical Center (LUMC, The Netherlands).

To investigate the association between MET immunohistochemical reactivity and survival, a tissue microarray (TMA) representing 240 FFPE primary oral or oropharyngeal SCC – surgically removed between 1996 and 2005 in the University Medical Center Utrecht (UMCU, the Netherlands) – was included in the study. Prior to the TMA's construction, hematoxylin and eosin (HE) sections – representing the selected cancers – and their corresponding FFPE tissue blocks were collected from the tissue archive of the department of pathology of the UMCU. Subsequently, a dedicated head and neck pathologist examined all HE slides with special attention to the following pathological characteristics: cancer type, differentiation grade, infiltration depth, growth pattern, perineural invasion, vasoinvasive growth, extranodal growth and bone invasion and selected vital cancer regions that were properly fixated for coring. In total, six tissue cores (0.6 mm diameter) were sampled from each cancer; three from the center and three from the periphery.

Using a microtome, 4 μm thick slices were cut from the FFPE cancer tissue and TMA blocks that were processed for immunohistochemistry.

### MET antibody validation cell line panel and culture conditions

Based on western blot results retrieved from the literature [21, 40, 54, 55] and datasheets provided with the purchased MET antibodies, a MET antibody validation cell line panel was developed. The panel consists of the following cell lines: LNCaP, SK-BR-3 (p145^MET^ negative according to the retrieved information), HeLa, HT-29, PC3, DU145 (p145^MET^ positive according to the retrieved information) and DU145#Sh167 (a *MET* silenced cell line). DU145#Sh167 was derived from DU145 by means of lentiviral infection as previously described [56]. Additional information is provided in Supplementary Table S1. All cell lines were obtained from different departments within the Erasmus Medical Center (EMC, Rotterdam, The Netherlands).

Cells were cultured at 37°C under 5% CO_2_ in DMEM/F12 (Life Technologies™) or RPMI 1640 medium (Life Technologies™), supplemented with varying percentages of Fetal Calf Serum (Life Technologies™) and 1% Penicillin/Streptomycin (Life Technologies™). Only DU145#Sh167 was grown in the presence of Puromycin (Sigma-Aldrich^®^; Zwijndrecht, The Netherlands). More information is indicated in Supplementary Table S6.

For all experiments (i.e., quantitative real-time PCR, western blot analysis, immunocytochemistry and immunohistochemistry) the cell lines were cultured as biological duplicates in view of independent validation of the results.

### RNA isolation, cDNA generation and quantitative real-time PCR

Cells were cultured in T25 flasks until 75-90% confluence was reached and harvested in TRIZol^®^ Reagent (Life Technologies™/Ambion^®^) for totRNA isolation according to the manufacturer's protocol. In total, 25 μg of totRNA was treated with RNase-free DNaseI (Life Technologies™/Ambion^®^) for 60 minutes at 37°C. Next, the totRNA was purified using the RNeasy mini kit (Qiagen; Venlo, The Netherlands) and eluted in 30 μL of DEPC treated H_2_O. Subsequently, cDNA was synthesized using SuperScript^®^ II Reverse Transcriptase (Life Technologies™/Invitrogen™) in accordance with the manufacturer's instructions.

Quantitative real-time PCR (qRT-PCR) reactions were performed in triplicate on two biological replicates (see above) of all cell lines included in the MET antibody validation panel using: TaqMan^®^ Gene Expression Assays against *MET* and *HPRT1* (Hs01565584_m1, Hs02800695_m1; Life Technologies™/Applied Biosystems^®^), TaqMan^®^ Universal Master Mix II, no UNG (Life Technologies™/Applied Biosystems^®^) and the 7500 Fast Real-Time PCR System (Life Technologies™/Applied Biosystems^®^) according to the manufacturer's protocol for 25 μL reaction volumes. To determine *MET* mRNA expression levels, a threshold (0.2) was set that falls in the exponential phase (4347825 Rev. F; Applied Biosystems^®^) of both target (*MET*) and endogenous control (*HPRT1*) across all samples. Subsequently, within sample normalization of *MET* expression was performed according to the relative standard curve method (4371095 Rev A; Applied Biosystems^®^).

### Protein isolation and western blot analysis

Cells were cultured in T75 flasks until 75-90% confluence was reached and harvested in lysis buffer (1% SDS, 10mM Tris, pH 7.5). Protein concentrations of the lysates were measured using the Pierce^®^ BCA Protein Assay Kit (Thermo Scientific; Bleiswijk, The Netherlands). Western blot analysis was performed on the Mini Protean II system (Bio-Rad; Veenendaal, The Netherlands). Cell extracts, mixed with 2x Laemmli Sample Buffer (Bio-Rad) including 2-mercaptoethanol, containing 18 μg of protein were separated on a 4-20% gradient polyacrylamide gel (Thermo Scientific). Subsequently, the protein fragments were transferred during 1.5 hour onto a PVDF membrane (GE Healthcare; Eindhoven, The Netherlands). After washing, the membrane was blocked for 1 hour with powder milk (5%; Royal FrieslandCampina; Amersfoort, the Netherlands) dissolved in PBS-Tween 0.1%. Next, the membranes were incubated O/N at 4°C with the primary MET antibodies (Supplementary Table S7). After washing multiple times with PBS-Tween 0.1%, the membranes were incubated for 1hr with the secondary ECL™ Anti-Rabbit IgG, HRP-Linked Whole Ab (1:5000; NA934V; GE Healthcare). After intensive washing with PBS-Tween 0.1%, SuperSignal^®^ West Femto Maximum Sensitivity Substrate (34095; Thermo Scientific) was used for detection. β-actin was visualized as loading control for each sample by means of the same protocol, using the primary mouse monoclonal antibody against b-actin (1:1000; clone AC-74; Sigma-Aldrich^®^) and the secondary Anti- Mouse IgG, HRP-linked whole Ab GPR (1:20000; NXA931; GE Healthcare).

### Immunocytochemical staining of MET antibody validation cell line panel

Cells were cultured on Nunc™ Lab-Tek™ Chamber slides (Thermo Scientific) until 60-90% confluence was reached. Subsequently, the cells were washed with cold PBS and immersed in fresh, cold Paraformaldehyde (4% v/w) for 20 min. for fixation. After blocking endogenous peroxidase activity with a 0.3% H_2_O_2_ solution for 5 minutes, slides were stained with the ABC procedure [57]. Endogenous biotin was blocked using the Avidin/Biotin blocking kit (SP-2001; Vector Laboratories Ltd; Peterborough, United Kingdom). Cells were incubated O/N at 4°C with the primary MET antibodies (Supplementary Table S7) and incubated for 30 minutes at room temperature with the secondary biotinylated polyclonal swine anti-rabbit antibody (1:150; E0431; Dako; Heverlee, Belgium). Signal amplification was performed with the VECTASTAIN^®^ Elite ABC system (PK-6100; Vector Laboratories Ltd) according to the manufacturer's protocol. Finally, horseradish peroxidase activity was visualized in 90 seconds with 3, 3′-diaminobenzidine (K3468; Dako) prepared in accordance with the manufacturer's instructions.

### Formalin fixation and paraffin embedding of MET antibody cell line panel

Cells were cultured in triplicate in T175 flasks until 75-90% confluence was reached. Subsequently, per cell line, all three cultures were harvested in a single volume of PBS (10 mL). After removing the PBS, the cells were fixed O/N at 4°C with 10% formalin (10 mL). After removing the fixative, the cells were resuspended in PBS (500 mL) to be transferred to a flat bottom embedding capsule (#70021; Electron Microscopy Sciences; Hatfield, PA, USA). Herein, after centrifugation and removal of the supernatant, the cells were dissolved in a PBS-agar (5.0%; Life Technologies™/Invitrogen™) solution (500 mL) of 56°C. Upon solidification on ice, the cell containing agar blocks were removed from their embedding capsules, transferred to cassettes and embedded in paraffin.

### Immunohistochemical staining of FFPE MET antibody validation cell line panel, whole tissue sections and TMA sections

After deparaffinization and rehydration, endogenous peroxidase activity was inactivated by incubating the sections in 3% H_2_O_2_ for 10 min. Subsequently, antigen retrieval was carried out by heating the sections under high pressure – up to 0.9 bar in case of the FFPE cell lines and up to 1.2 bar in case of the FFPE tissues – in Tris-EGTA buffer (0.01 M Tris, 0.001 M EGTA, pH 9.0).

After antigen retrieval, the tissue sections were stained with the ABC procedure using an almost identical protocol applied for the immunochemical staining of the MET antibody validation cell line panel. However, in contrast to the immunocytochemical procedure, the sections were incubated with 0.22% bovine serum albumin solution (A7034; Sigma-Aldrich^®^) in 1X PBS for 7 min. after blocking endogenous biotin to reduce non-specific background staining. Instead of 90 seconds, horseradish peroxidase activity was visualized in 2x 5 minutes. The used primary antibody titers are summarized in Supplementary Table S7 and the sections were counterstained with haematoxylin before mounting them with a coverslip.

### Evaluation of MET immunocyto & immunohistochemical staining

#### Cells included in the antibody validation panel and whole tissue sections of oral SCC

*D1C2, SP44, CVD13, C-12 and C-28*. Staining intensities were evaluated for nuclear, cytoplasmic and membranous immunoreactivity on a scale from 0 to 3. For nuclear and cytoplasmic immunoreactivity this means: 0 = no staining, 1 = weak staining, 2 = moderate staining and 3 = strong staining. For membranous immunoreactivity this means: 0 = no staining, 1 = weak complete or incomplete membranous staining, 2 = moderate complete membranous staining and 3= strong complete membranous staining. Representative images of nuclear, cytoplasmic and membranous staining intensities are depicted in Supplementary Figure S1D.

The final immunoreactivity score was defined as the maximum observed staining intensity involving at least 10% of the cancer cells. Scores were given separately for the nucleus, cytoplasm and membrane.

The cell lines cultured on chamber slides were evaluated in areas with equal cell density and similar morphology to assure comparability.

In case of the whole tissue sections, well-differentiated cancer cells that show no nuclei were omitted during scoring.

Two observers (SK and MDH) simultaneously assessed the immunoreactivity scores.

#### TMA representing a cohort of oral and oropharyngeal SCC

*D1C2*. Evaluation of MET staining intensities was restricted to membranous immunoreactivity as defined above. Cancer cells showing no (0) to weak (1) membranous immunoreactivity were assessed as negative for MET, while cancer cells showing moderate (2) to strong (3) membranous immunoreactivity were assessed as positive for MET. Moreover, seemingly suboptimal fixed cores and well-differentiated cancer cells that show no nuclei were omitted during scoring.

Two observers (SW and MDH) independently assessed the percentage of MET positive cancer cells for each tissue core (Supplementary Figure S1E). Agreement was evaluated by means of a Bland and Altman diagram (Supplementary Figure S1F). In case of discordant scores, both observers simultaneously reassessed their counts after deliberation (Supplementary Figure S1G).

If more than one core was available for evaluation per cancer region (center and periphery, see patient material), the average percentage of MET positive tumor cells was calculated. Evaluation of MET immunoreactivity in both cancer regions was possible in 183 out of the 240 tumors (76.3%) that are represented on the TMA (see patient material). Of these 183 tumors, only 4 oropharyngeal SCC were HPV-16 positive. Seen the low number, HPV-16 positive tumors were omitted from further analysis. The remaining patient population (*n* = 179) – evaluated for MET immunoreactivity in both the center and periphery – consisted of 114 males (63.7%) and comprised 131 advanced stage (III-IV) cancers (73.2%). Further baseline characteristics are indicated in Table 2.

To investigate the consistency of MET immunoreactivity within the cancer center and periphery, we analyzed the Intraclass Correlation Coefficient (ICC) between the three scored cores per tumor region. The ICC is a descriptive statistic which describes how strongly different quantitative measures resemble each other, in this case multiple cores of the same tumor. An ICC < 0 reflects ‘poor’, 0 to 0.20 ‘slight’, 0.21 to 0.4 ‘fair’, 0.41 to 0.60 ‘moderate’, 0.61 to 0.8 ‘substantial’, and above 0.81 ‘almost perfect’ reliability of the measurement. Any measure should have an ICC of at least 0.6 to be useful with regard to reliability of the result. The 95% confidence intervals were indicated between brackets. Calculations were done with SPSS Statistics (version 21; IBM; Armonk, New York).

### Survival analysis

Overall survival (OS) was defined as the time in months from the date of primary surgery to: the date of death due to any cause or the cutoff time (set at 60 months). Disease free survival (DFS) was defined as the time in months from the date of primary surgery to: the date of first evidence of any disease (local, regional, distant or secondary primary) progression, the date of death due to any cause or the cutoff time (set at 60 months). Individuals who were: lost to follow-up or survived beyond the cutoff time, were considered as censored observations. MET immunoreactivity OS as well as DFS curves were calculated by means of the Kaplan-Meier method and significance of differences in survival times was assessed with the log-rank test. Univariable as well as multivariable Cox proportional hazards regression models were used to evaluate the prognostic value of MET immunoreactivity, demographical, clinical, and histopathological patient characteristics. Variables significantly associated with OS and DFS as well as potential confounders were included in backward selection procedures to select the final models. Calculations were done with SPSS Statistics (version 21; IBM) and R version 2.15.3 (version http://www.r-project.org). Unless otherwise mentioned, statistical significance was set at *p* < 0.05.

## SUPPLEMENTARY FIGURES AND TABLES


