# Supplementary material for: Absent and abundant MET immunoreactivity is associated with poor prognosis of patients with oral and oropharyngeal squamous cell carcinoma
Source: Oncotarget. 2016 Feb 20;7(11):13167–81. doi: 10.18632/oncotarget.7534 (PMC4914349; doi:10.18632/oncotarget.7534)
Supplement: Supplementary file 1 [file oncotarget-07-13167-s001.pdf]

SUPPLEMENTARY FIGURES AND TABLES

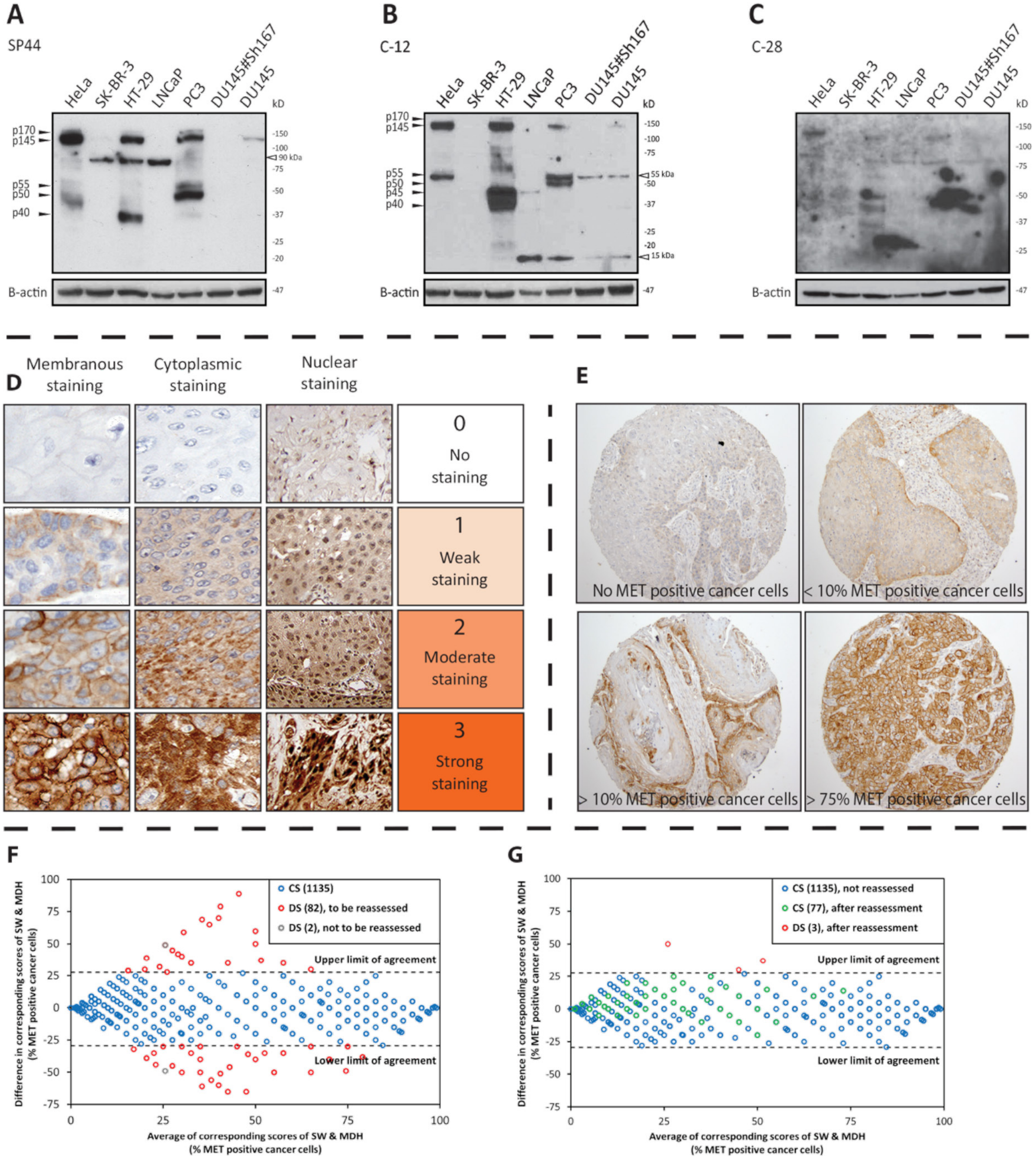

Supplementary Figure S1: Supplementary figures concerning MET immunoreactivity and antibody validation. A through C. immunoreactivity across the MET antibody validation cell line panel under reducing conditions observed with SP44 (A), C-12 (B) and C-28 (C).The immunoblots generated with SP44 and C-12 show MET specific as well as nonspecific protein bands. The MET specific protein bands are labelled on the left of each immunoblot. The MET nonspecific protein bands are labelled on the right of each immunoblot. For further information concerning the MET specific protein bands, the reader is referred to Supplementary table 2. The immunoblot generated with C-28 was found too poor to evaluate. **D.** images representing membranous, cytoplasmic and nuclear staining intensities. **E.** images representing percentages of MET positive cancer cells – membranous staining intensities 2-3 – observed with D1C2 across tissue cores sampled on the TMA. **F. & G.** Bland and Altman plots showing the limits of agreement for concordant corresponding tissue core scores of SW & MDH. The limits of agreement are the upper and lower limits of the interval $d \pm 1.96s_d$ where d is the mean of the differences between corresponding observations of SW and MDH and s_d its standard deviation. Before reassessment there were 82 – depicted between brackets – discordant scores (red circles) (F). After reassessment this number is reduced to 3 (G). Abbreviations: CS, Concordant Score; DS, Discordant Score.

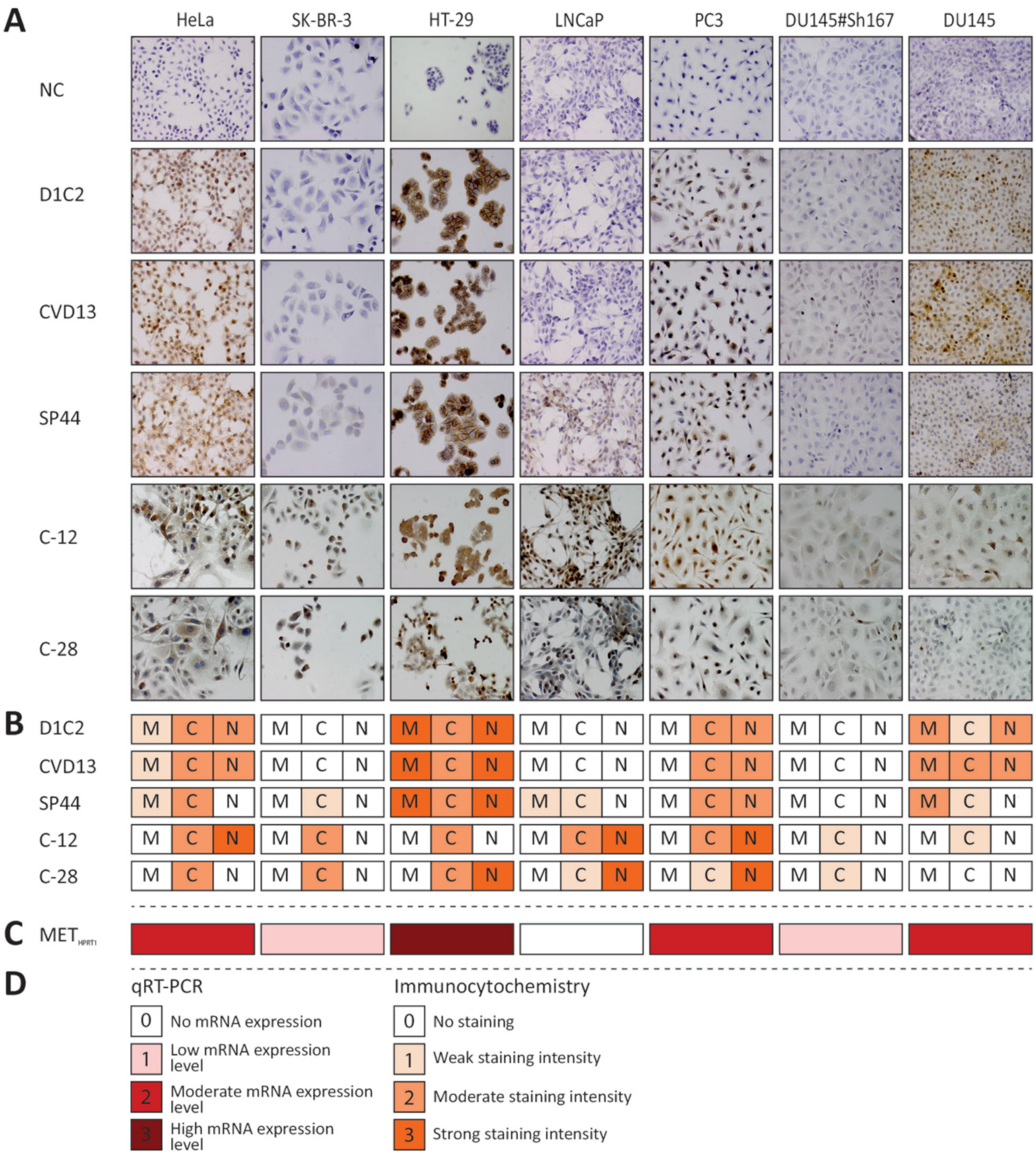

Supplementary Figure S2: Immunoreactivities of the five tested C-terminal MET antibodies under native conditions in respect to *MET* mRNA expression levels across the antibody validation cell line panel. A. representative immunoreactivities of all antibodies with all cell lines and the corresponding negative controls (NCs). **B.** membranous (M), cytoplasmic **C.** and nuclear (N) immunocytochemical reactivity. (C) qRT-PCR results showing average *MET* fluorescence standardized to average *HPRT1* fluorescence. **D.** Legend for observed mRNA levels and immunocytochemical reactivities. It should be noted that pictures used to present the immunoreactivities obtained with D1C2 and CVD13 are the same as those presented in Figure 1C.

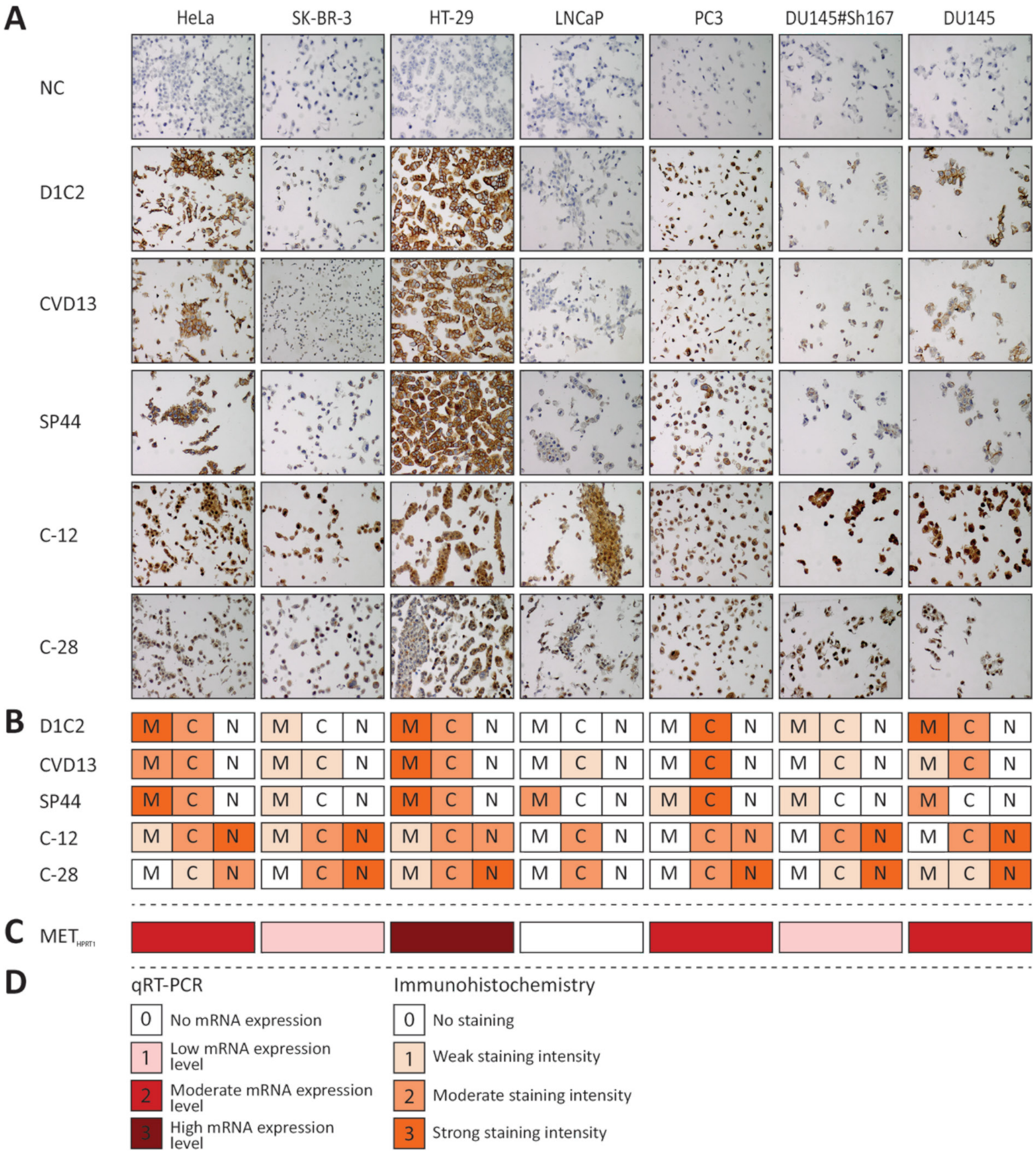

Supplementary Figure S3: Immunoreactivities of the five tested C-terminal MET antibodies under FFPE conditions in respect to *MET* mRNA expression levels of the antibody validation cell line panel. A. representative immunoreactivities of all antibodies with all cell lines and the corresponding negative controls (NCs). **B.** membranous (M), cytoplasmic **C.** and nuclear (N) immunohistochemical reactivity. (C) qRT-PCR results showing average *MET* fluorescence standardized to average *HPRT1* fluorescence. **D.** Legend for observed mRNA expression levels and immunohistochemical reactivities. It should be noted that pictures used to present the immunoreactivities obtained with D1C2 and CVD13 are the same as those presented in Figure 1D.

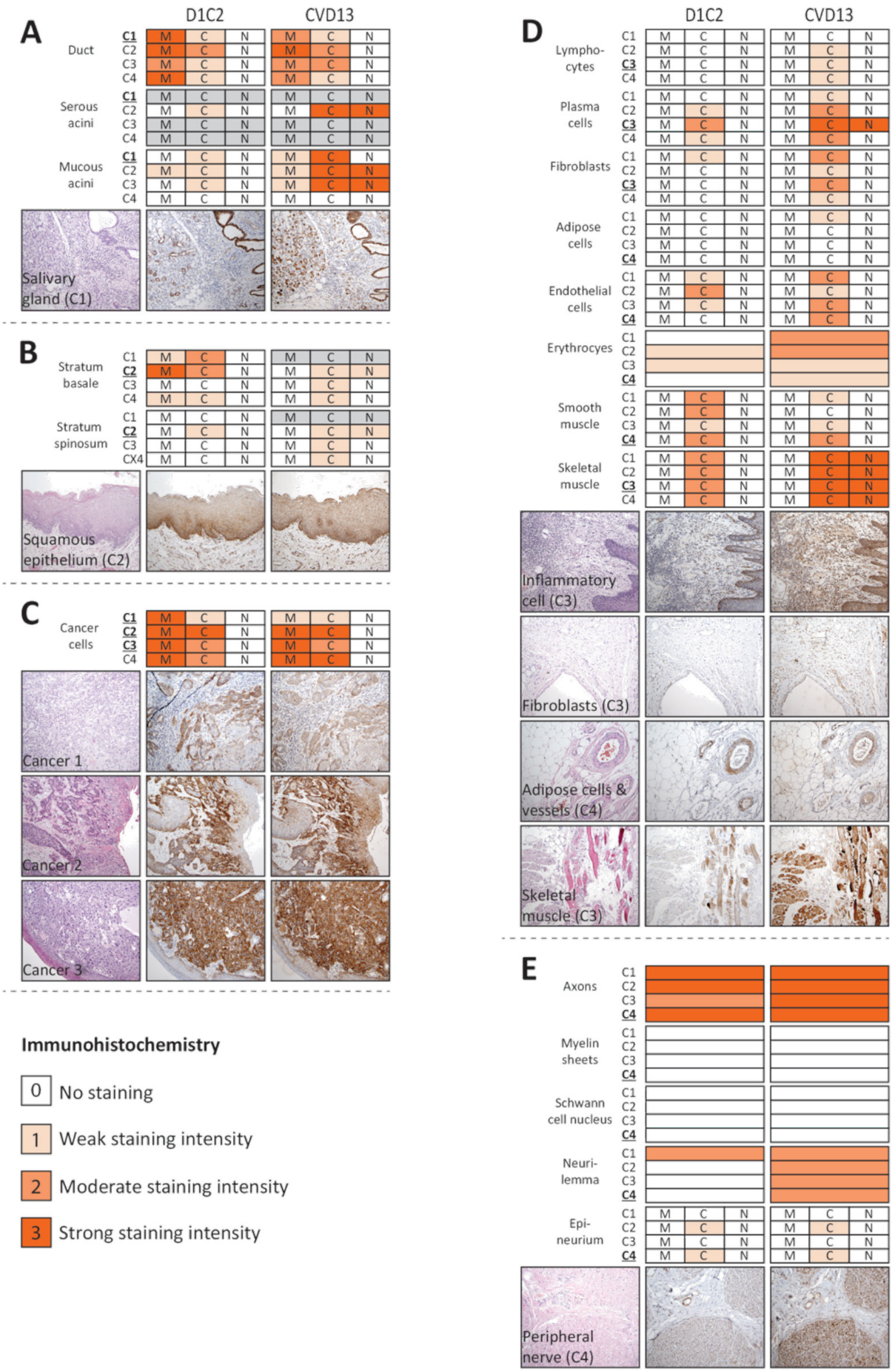

Supplementary Figure S4: Immunohistochemical reactivity of D1C2 and CVD13 with whole tissue sections of FFPE oral SCC. Membranous, cytoplasmic and nuclear immunoreactivities were determined for the different tissues structures and cell types observed in 4 cancer specimens (C1-4). **A.** salivary gland. **B.** squamous epithelium. **C.** cancer cells. **D.** connective tissue, vessels and muscle. **E.** peripheral nerve. The observed immunoreactivities – per tissues structure or cell type – were illustrated with representative photographs taken from the evaluated cancers.

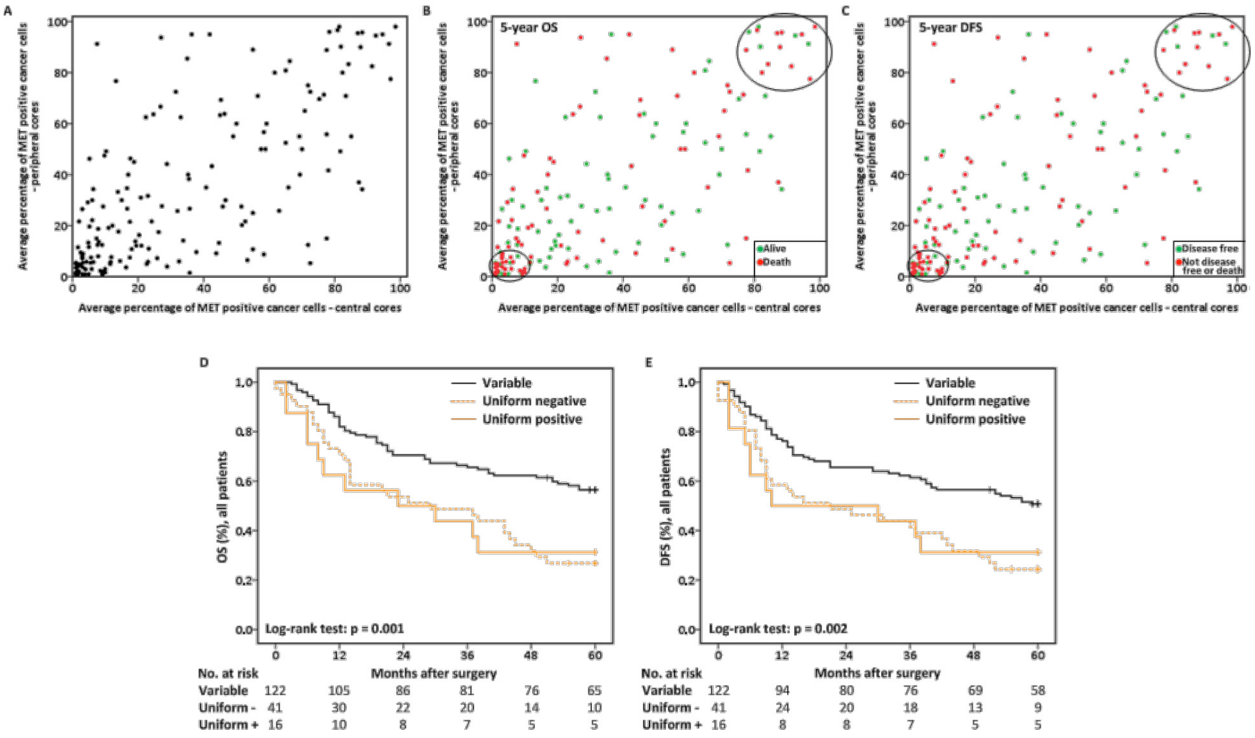

Supplementary Figure S5: Evaluation of MET immunoreactivity using a scatter plot in view of survival analysis. A through C. The scatter plot depicted under (A) – used to characterize MET immunoreactivity across cancer surfaces – reveals constant and variable membranous MET staining across cancer surfaces. The scatter plots depicted under (B & C) – used to examine the relation between the pattern of MET immunoreactivity and survival (green dot: alive; red dot: deceased) – reveal that the relative number of events (i.e. OS or DFS) within the clusters (dots within the circles) that depict uniform staining (negative or positive) is higher compared to the relative number of events outside them for both 5-year OS (B) and DFS (C). **Kaplan-Meier curves and the No. of patients at risk. D.** 5-year OS **& E.** 5-year DFS for all patients, stratified by MET staining pattern.

Supplementary Table S1: Cell lines included in the MET antibody validation panel and their properties

Cell line	Organism	Organ	Metastatic site	Disease	Morphology	Expected p145^{MET} status
HeLa	Homo sapiens	Cervix	Not applicable	Adenocarcinoma	Epithelial	Positive [1]**, ***
SK-BR-3*	Homo sapiens	Mammary gland; breast	Pleural effusion	Adenocarcinoma	Epithelial	Negative [2]***
HT-29	Homo sapiens	Colon; colorectal	Not applicable	Adenocarcinoma	Epithelial	Positive [3]**
LNCaP*	Homo sapiens	Prostate	Left supraclavicular lymph node	Carcinoma	Epithelial	Negative [4]**, ***
PC3*	Homo sapiens	Prostate	Bone	Adenocarcinoma	Epithelial	Positive [4]**, ***
DU145*	Homo sapiens	Prostate	Brain	Carcinoma	Epithelial	Positive [4]**, ***

* Derived from metastatic site.
** p145^{MET} is detected on cell lysate by means of western blot using a MET antibody directed against the extracellular domain of the protein.
*** p145^{MET} is detected on cell lysate by means of western blot using a MET antibody directed against the intracellular domain of the protein.

Supplementary Table S2: Anticipated MET protein products and C-terminal fragments

Products and fragments	Description	Used abbreviations Figure 1B, Supplementary figures 1A & 1B	References
$p170^{MET}$	Single chain MET precursor	p170	[3]
$p145^{MET}$	MET full-length β-subunit	p145	[3]
$p95^{MET}$	Possible C-terminal fragment resulting after ectodomain shedding	p95	[5]
$p90^{MET}$	Possible C-terminal fragment resulting after ectodomain shedding	p90	[5]
$p70^{MET}$	Possible C-terminal fragment resulting after ectodomain shedding	p70	[3] [5]
$p60^{MET}$	Possible C-terminal fragment resulting after ectodomain shedding	p60	[3] [5]
$p55^{MET}$	Membrane-anchored C-terminal fragment	p55	[6]
$p50^{MET}$	Intracellular domain of MET	p50	[1]
$p45^{MET}$	Possible C-terminal fragment resulting after ectodomain shedding	p45	[5]
$p40^{MET}$	Proapoptotic intracellular 40 kDa C-terminal fragment	p40	[7] [8]

Supplementary Table S3: The effect of baseline characteristics on 5-year OS and DFS

Explantory variable	No. of patients		5-year OS			5-year DFS		
	#	%	HR	95% CI	p-value	HR	95% CI	p-value
Sex								
Male	114	63.69						
Female	65	36.31	1.181	0.781 – 1.787	0.431	1.249	0.841 – 1.857	0.271
Age at diagnosis (years)								
Mean (range)	62.97 (34 – 87)		1.017	0.998 – 1.035	0.075	1.019	1.001 – 1.037	**0.039**
Smoking								
No	60	33.52						
Yes	117	65.36	1.289	0.823 – 2.019	0.268	1.200	0.785 – 1.833	0.400
Missing	2	1.12						

(*Continued*)

Explantory variable	No. of patients		5-year OS			5-year DFS		
	#	%	HR	95% CI	p-value	HR	95% CI	p-value
Alcohol								
No	81	45.25						
Yes	96	53.63	0.978	0.649 – 1.475	0.917	0.859	0.580 – 1.272	0.449
Missing	2	1.12						
Site								
Oral cavity	157	87.71						
Oropharynx	22	12.29	0.865	0.461 – 1.623	0.653	0.882	0.483 – 1.611	0.682
cT-stage								
cT1-2	95	53.07						
cT2-3	84	46.93	2.005	1.329 – 3.027	**0.001**	1.776	1.200 – 2.629	**0.004**
cN-stage								
cN0	113	63.13						
cN1-3	66	36.87	2.998	1.991 – 4.514	**< 0.001**	2.520	1.702 – 3.729	**< 0.001**
pT-stage								
pT1-2	91	50.84						
pT2-3	88	49.16	2.218	1.457 – 3.377	**< 0.001**	1.909	1.283 – 2.841	**0.001**
pN-stage								
pN0	75	41.90						
pN1-3	104	58.10	2.035	1.318 – 3.144	**0.001**	1.867	1.239 – 2.814	**0.003**
Infiltration depth								
<4.0 mm	9	5.03						
≥4.0 mm	170	94.97	1.778	0.563 – 5.616	0.327	2.087	0.662 – 6.584	0.209
Differentiation grade								
Good-moderate	141	78.77						
Poor	38	21.23	1.743	1.102 – 2.756	**0.017**	1.541	0.981 – 2.421	0.061
Vasoinvasive growth								
Absent	136	75.98						
Present	39	21.79	1.785	1.136 – 2.804	**0.012**	1.557	0.999 – 2.427	0.05
Missing	4	2.23						
Bone invasion								
Absent OR no bone present	140	78.21						
Present	39	21.79	1.526	0.965 – 2.412	0.071	1.327	0.845 – 2.085	0.219

(*Continued*)

Explantory variable	No. of patients		5-year OS			5-year DFS		
	#	%	HR	95% CI	p-value	HR	95% CI	p-value
Perineural invasion								
Absent	98	54.75						
Present	73	40.78	1.244	0.819 – 1.890	0.306	1.168	0.781 – 1.746	0.450
Missing	8	4.47						
Extranodal growth								
Absent OR pN0	124	69.27						
Present	54	30.17	3.927	2.595 – 5.942	**< 0.001**	3.616	2.423 – 5.396	**< 0.001**
Missing	1	0.56						
Growth pattern								
Cohesive	36	20.11						
Non-cohesive	142	79.33	1.613	0.928 – 2.802	0.09	1.398	0.839 – 2.328	0.198
Missing	1	0.56						
Treatment								
Surgery	50	27.93						
Surgery and (C) XRT	128	71.51	1.537	0.936 – 2.523	0.089	1.313	0.830 – 2.078	0.245
Missing	1	0.56						

Abbreviations: OS, Overall Survival; DFS, Disease Free Survival; HR, Hazard Ratio; CI, Confidence Interval; (C)XRT, (chemo)radiotherapy. Bold values highlight statistical significance.

Supplementary Table S4: Evaluation of possible confounders and significant statistical interaction for 5-year OS

Explanatory variable	5-year OS				
	Confounder			Effect modifier	
	b_1 eq. 1	b_1 eq. 2	Variation b_1's (%)	$\exp\{b_3\}$ eq. 3	*p*-value
Sex	0.78	0.77	1.28	0.61	0.25
Age at diagnosis (years)	0.78	0.76	3.19	0.99	0.71
Smoking	0.80	0.78	1.88	1.21	0.69
Alcohol	0.80	0.81	−1.25	0.83	0.66
Site	0.78	0.78	0.38	0.39	0.24
cT-stage	0.78	0.67	14.43	0.63	0.27
cN-stage	0.78	0.74	5.87	0.58	0.19
pT-stage	0.78	0.69	12.26	0.70	0.41
pN-stage	0.78	0.80	−1.92	0.89	0.78
Infiltration depth	0.78	0.78	1.02	0.25	0.27
Differentiation grade	0.78	0.76	2.43	1.02	0.98
Vasoinvasive growth	0.81	0.74	8.17	0.30	**0.01**
Bone invasion	0.78	0.74	6.00	0.86	0.75
Perineural invasion	0.83	0.92	−11.86	1.73	0.21
Extranodal growth	0.77	0.60	22.15	0.54	0.14
Growth pattern	0.82	0.87	−6.50	1.08	0.90
Postoperative (C)XRT	0.80	0.83	−4.65	0.36	0.06

Abbreviations: OS, Overall Survival; (C)XRT, (chemo)radiotherapy; eq., equation. Bold values highlight statistical significance.

Supplementary Table S5: Evaluation of possible confounders and significant statistical interaction for 5-year DFS

Explanatory variable	5-year DFS				
	Confounder			Effect modifier	
	b_1 eq. 1	b_1 eq. 2	Variation b_1's (%)	$\exp\{b_3\}$ eq. 3	p-value
Sex	0.68	0.66	2.65	0.52	0.11
Age at diagnosis (years)	0.68	0.65	5.15	0.98	0.31
Smoking	0.68	0.67	1.47	1.54	0.35
Alcohol	0.68	0.70	–1.90	1.20	0.65
Site	0.68	0.68	0.44	0.41	0.27
cT-stage	0.68	0.60	11.91	0.89	0.78
cN-stage	0.68	0.67	1.47	0.91	0.81
pT-stage	0.68	0.62	8.68	0.89	0.77
pN-stage	0.68	0.70	–2.65	0.98	0.96
Infiltration depth	0.68	0.67	0.88	0.26	0.28
Differentiation grade	0.68	0.67	2.21	1.14	0.78
Vasoinvasive growth	0.70	0.66	6.27	0.37	**0.03**
Bone invasion	0.68	0.66	3.53	1.01	0.98
Perineural invasion	0.71	0.79	–11.13	1.93	0.12
Extranodal growth	0.67	0.52	23.02	0.61	0.22
Growth pattern	0.68	0.72	–6.16	1.45	0.48
Postoperative (C)XRT	0.69	0.72	–4.64	0.50	0.16

Abbreviations: DFS, Disease Free Survival; (C)XRT, (chemo)radiotherapy; eq., equation. Bold values highlight statistical significance.
SUPPOSE logistic regression equations
$\lambda_i(t) = \lambda_0(t)\exp\{b_1x_1\}$ [1]
$\lambda_i(t) = \lambda_0(t)\exp\{b_1x_1 + b_2x_2\}$ [2]
$\lambda_i(t) = \lambda_0(t)\exp\{b_1x_1 + b_2x_2 + b_3x_1.x_2\}$ [3]
WITH
x_1: MET staining pattern
x_2: to be investigated baseline variable
THEN
x_1 and x_2 are confounders when there is more than 10% variation between the b_1's from equation 1 and 2
Statistical interaction occurs between x_1 and x_2 if $\exp\{b_3\}$ significantly contributes to equation 3

Supplementary Table S6: MET antibody validation cell line panel culture conditions

Cell line	T (°C)	CO_2 (%)	Medium	Supplements
HeLa	37	5	DMEM/F12 (Life Technologies™)	10% Fetal Calf Serum (Life Technologies™) and 1% Penicillin/Streptomycin (Life Technologies™)
SK-BR-3	37	5	RPMI 1640 (Life Technologies™)	10% Fetal Calf Serum (Life Technologies™) and 1% Penicillin/Streptomycin (Life Technologies™)
HT-29	37	5	RPMI 1640 (Life Technologies™)	5% Fetal Calf Serum (Life Technologies™) and 1% Penicillin/Streptomycin (Life Technologies™)
LNCaP	37	5	DMEM/F12 (Life Technologies™)	10% Fetal Calf Serum (Life Technologies™) and 1% Penicillin/Streptomycin (Life Technologies™)
PC3	37	5	DMEM/F12 (Life Technologies™)	10% Fetal Calf Serum (Life Technologies™) and 1% Penicillin/Streptomycin (Life Technologies™)
DU145#Sh167	37	5	RPMI 1640 (Life Technologies™)	5% Fetal Calf Serum (Life Technologies™), 1% Penicillin/Streptomycin (Life Technologies™) and 0.5 µg/mL Puromycin (Sigma-Aldrich®)
DU145	37	5	RPMI 1640 (Life Technologies™)	5% Fetal Calf Serum (Life Technologies™) and 1% Penicillin/Streptomycin (Life Technologies™)

Supplementary Table S7: MET antibody titers used during different applications

Clone	Western blot	Immunocytochemistry	IHC FFPE cell lines	IHC FFPE tissues
D1C2	1:1000	1:1000	1:400	1:100
SP44	1:1000	1:1000	1:800	1:200
CVD13	1:1000	1:1000	1:800	1:200
C-12	1:1000	1:1000	1:800	1:200
C-28	1:500	1:500	1:400	1:100

Abbreviation: IHC, immunohistochemistry.

REFERENCES

1. Foveau B, Ancot F, Leroy C, Petrelli A, Reiss K, Vingtdeux V, Giordano S, Fafeur V and Tulasne D. Down-regulation of the met receptor tyrosine kinase by presenilin-dependent regulated intramembrane proteolysis. Mol Biol Cell. 2009; 20:2495–2507.
2. Brusevold IJ, Soland TM, Khuu C, Christoffersen T and Bryne M. Nuclear and cytoplasmic expression of Met in oral squamous cell carcinoma and in an organotypic oral cancer model. Eur J Oral Sci. 2010; 118:342–349.
3. Prat M, Crepaldi T, Gandino L, Giordano S, Longati P and Comoglio P. C-terminal truncated forms of Met, the hepatocyte growth factor receptor. Mol Cell Biol. 1991; 11:5954–5962.
4. Tate A, Isotani S, Bradley MJ, Sikes RA, Davis R, Chung LW and Edlund M. Met-Independent Hepatocyte Growth Factor-mediated regulation of cell adhesion in human prostate cancer cells. BMC Cancer. 2006; 6:197.
5. Athauda G, Giubellino A, Coleman JA, Horak C, Steeg PS, Lee MJ, Trepel J, Wimberly J, Sun J, Coxon A, Burgess TL and Bottaro DP. c-Met ectodomain shedding rate correlates with malignant potential. Clin Cancer Res. 2006; 12:4154–4162.
6. Ancot F, Leroy C, Muharram G, Lefebvre J, Vicogne J, Lemiere A, Kherrouche Z, Foveau B, Pourtier A, Melnyk O, Giordano S, Chotteau-Lelievre A and Tulasne D. Shedding-generated Met receptor fragments can be routed to either the proteasomal or the lysosomal degradation pathway. Traffic. 2012; 13:1261–1272.
7. Foveau B, Leroy C, Ancot F, Deheuninck J, Ji Z, Fafeur V and Tulasne D. Amplification of apoptosis through sequential caspase cleavage of the MET tyrosine kinase receptor. Cell Death Differ. 2007; 14:752–764.
8. Tulasne D, Deheuninck J, Lourenco FC, Lamballe F, Ji Z, Leroy C, Puchois E, Moumen A, Maina F, Mehlen P and Fafeur V. Proapoptotic function of the MET tyrosine kinase receptor through caspase cleavage. Mol Cell Biol. 2004; 24:10328–10339.
